# Supplementary material for: The oncolytic virus Delta-24-RGD elicits an antitumor effect in pediatric glioma and DIPG mouse models
Source: Nat Commun. 2019 May 28;10:2235. doi: 10.1038/s41467-019-10043-0 (PMC6538754; doi:10.1038/s41467-019-10043-0)
Supplement: Supplementary file 1 — Supplementary Information [file 41467_2019_10043_MOESM1_ESM.pdf]

Supplementary Tables

Supplementary Table 1. Characteristics of the cell lines used in this study

Cell culture	Histone 3 mutation	Sex	Age	Reference
TP54	H3.3K27M	Male	9 years	1, 2
TP80	H3.3K27M	Female	5 years	1
TP83	H3.3K27M	Female	5 years	1
TP84	H3.3K27M	Male	9 years	1
SU-DIPG IV	H3.1K27M	Female	2 years	3
JHH-DIPG I	H3.3K27M	Male	8 years	4
JHH-DIPG-16A	ND	Female	4 years	5
CHLA-200	H3WT	Male	12 years	6
CHLA-03-AA	H3WT	Female	9 years	7
SJ-GBM2	H3WT	Female	4, 2 years	8-10
SF-188	H3WT	Male	8 years	11-13
PBT-24	H3WT	Male	13 years	NP

ND= Non Determined; NP= Non-Published yet

Supplementary Table 2. Evaluation of Delta-24-RGD dose-escalation injection toxicity following administration into the pons. Toxicity was evaluated by the evaluation of different symptoms including paresia, gate abnormality, difficulty of access to food or water, weight loss of >20% and the death of the animal after viral or PBS injection.

Treatment (Dose Escalation)	Toxicity/Animal survival
PBS (N=9)	0/9
10^6 pfu (N=10)	0/10
$5x10^6$ pfu (N=10)	0/10
10^7 pfu (N=10)	0/10
$5x10^7$ pfu (N=10)	0/10
10^8 pfu (N=10)	0/10

Supplementary Table 3. Evaluation of Delta-24-RGD injection toxicity following administration of one versus three viral injections. Toxicity was evaluated by the evaluation of different symptoms including paresia, gate abnormality, difficulty of access to food or water, weight loss of >20% and the death of the animal after viral or PBS injection.

Treatment	Injections	Toxicity
PBS (N=9)	3	0/10
10^8 pfu (N=10)	1	0/10
10^8 pfu (N=10)	3	0/10

Supplementary methods

Flow cytometry

Infectivity was assessed by quantification of GFP positive cells. Cells were infected at 10 and 100 MOIs with a no replicative adenovirus expressing GFP (Ad5-GFP-RGD). Infected and non-infected cells were recollected 36 hours later and they were analyzed by flow cytometry. Green (510–530 nm) fluorescence emission from 10,000 cells illuminated with blue (488 nm) excitation light was measured with a FACSCalibur (Becton Dickinson [BD], San Jose, CA, USA) using the BD CellQuest software.

The analysis of viral receptor ($\alpha\nu\beta_3$, $\alpha\nu\beta_5$ integrin and CAR) expression in the membrane of pediatric glioma cells were performed by flow citometry. 300000 cells were recollected in 100ul of PBS-BSA buffer. Primary $\alpha\nu\beta_3$ (Anti- $\alpha\nu\beta_3$ antibody clone 23C6, Merck KGaA, Darmstadt, Germany), $\alpha\nu\beta_5$ integrin-FITC (Anti-$\alpha\nu\beta_5$ antibody, clone P1F6, Merck KGaA, Darmstadt, Germany) and CAR (Anti-CAR antibody, clone RmcB, Merck KGaA, Darmstadt, Germany) antibody were add to cell suspension and it was incubated during 1hour at 4ºC. Stained cells were washed with BSA-PBS buffer. CAR and $\alpha\nu\beta_3$integrin were also incubated with anti IgG_1- FITC antibody (Santa Cruz Bio-technology, Santa Cruz, CA) for 30 minutes at 4ºC, avoiding direct light. Fluorescence emission of 10,000 cells was measured with FACSCalibur cytometer using BD CellQuest software (Becton Dickinson [BD], San Jose, CA, USA).

Tumor establishment procedure

Under aseptic conditions and with all materials sterilized according to standard techniques, mice of four weeks of age were anesthetized by intraperitoneal injection with ketamine and xilacyne solution. The animal heads were supported by a couple of rolled gauzes so that when the screw was inserted, pressure applied over neck and head structures was better tolerated by the animal.

We prepared mice head skin with povidone iodine solution prior to make a 5 mm-long lineal skin incision with 23-size scalpel and expose skull sutures. We first made a small mark according to the coordinates with a small 15-gauge needle which was subsequently widened with a hand-controlled twist drill which penetrates the skull. Next, we introduced the screw with its specific screwdriver by applying slight pressure throughout the previous twist hole. The coordinates for generation of DIPG tumors are 1.0 mm right to lambda and just posterior (0.8 mm) to lambdoid suture, while coordinates for pHGG tumors are from bregma (intersection between coronal and sagittal suture) 1 mm anterior and 2.5mm to the right. Thereafter the needle of Hamilton syringe is slowly introduced into the hole by applying gentle pressure until the sleeve/cuff from the syringe reaches the screw surface. The desired depth to reach brainstem is 6.5 mm and depth for hemispheric tumors (pHGG) is 3.5mm. Cell suspension was carefully injected using an infusion pump (Harvard Apparatus) over 20 minutes.

Supplementary References

1.- Thirant C, Bessette B, Varlet P, Puget S, Cadusseau J, Tavares Sdos R, Studler JM, Silvestre DC, Susini A, Villa C, Miquel C, Bogeas A, Surena AL, Dias-Morais A, Léonard N, Pflumio F, Bièche I, Boussin FD, Sainte-Rose C, Grill J, Daumas-Duport C, Chneiweiss H, Junier MP. Clinical relevance of tumor cells with stem-like properties in pediatric brain tumors. PLoS One. 6:e16375. doi: 10.1371/journal.pone.0016375 (2011)

2.- El-Habr EA, Dubois LG, Burel-Vandenbos F, Bogeas A, Lipecka J, Turchi L, Lejeune FX, Coehlo PL, Yamaki T, Wittmann BM, Fareh M, Mahfoudhi E, Janin M, Narayanan A, Morvan-Dubois G, Schmitt C, Verreault M, Oliver L, Sharif A, Pallud J, Devaux B, Puget S, Korkolopoulou P, Varlet P, Ottolenghi C, Plo I, Moura-Neto V, Virolle T, Chneiweiss H, Junier MP. A driver role for GABA metabolism in controlling stem and proliferative cell state through GHB production in glioma. Acta Neuropathol. 133:645-660 (2017)

3.- Grasso C.S., Tang Y., Truffaux N., Berlow N.E., Liu L., Debily M.-A., Quist M.J., Davis L.E., Huang E.C., Woo P.J., Ponnuswami A., Chen S., Johung T.B., Sun W., Kogiso M., Du Y., Qi L., Huang Y., Hutt-Cabezas M., Warren K.E., Le Dret L., Meltzer P.S., Mao H., Quezado M., van Vuurden D.G., Abraham J., Fouladi M., Svalina M.N., Wang N., Hawkins C., Nazarian J., Alonso M.M., Raabe E.H., Hulleman E., Spellman P.T., Li X.-N., Keller C., Pal R., Grill J., Monje M. Functionally defined therapeutic targets in diffuse intrinsic pontine glioma. Nat. Med. 21:555-559(2015)

4.- Taylor I.C., Hutt-Cabezas M., Brandt W.D., Kambhampati M., Nazarian J., Chang H.T., Warren K.E., Eberhart C.G., Raabe E.H. Disrupting NOTCH slows diffuse intrinsic pontine glioma growth, enhances radiation sensitivity, and shows combinatorial efficacy with bromodomain inhibition. J. Neuropathol. Exp. Neurol. 74:778-790(2015)

5.- Shah S., Rubens J., Eberhart C., and Raabe E.H. DIPG-62 Carboplatin synergizes with bcl-2 inhbitor to potentiate killing of DIPG cells. *NeuroOncol*, 20, Issue suppl_2, 22, Pages i61. https://doi.org/10.1093/neuonc/noy059.155

6.- Xu J., Erdreich-Epstein A., Gonzalez-Gomez I., Melendez E.Y., Smbatyan G., Moats R.A., Rosol M., Biegel J.A., Reynolds C.P. Novel cell lines established from pediatric brain tumors. J. Neurooncol. 107:269-280 (2012)

7.- Xu J., Margol A.S., Asgharzadeh S., Erdreich-Epstein A. Pediatric brain tumor cell lines. J. Cell. Biochem. 116:218-224 (2015)

8.- Houghton P.J., Cheshire P.J., Hallman J.D. II, Lutz L., Friedman H.S., Danks M.K., Houghton J.A. Efficacy of topoisomerase I inhibitors, topotecan and irinotecan, administered at low dose levels in protracted schedules to mice bearing xenografts of human tumors. Cancer Chemother. Pharmacol. 36:393-403(1995)

9.- Sharif T.R., Luo W., Sharif M. Functional expression of bombesin receptor in most adult and pediatric human glioblastoma cell lines; role in mitogenesis and in stimulating the mitogen-activated protein kinase pathway. Mol. Cell. Endocrinol. 130:119-130(1997)

10.- Kang M.H., Smith M.A., Morton C.L., Keshelava N., Houghton P.J., Reynolds C.P. National Cancer Institute pediatric preclinical testing program: model description for in vitro cytotoxicity testing. Pediatr. Blood Cancer 56:239-249(2011)

11.- Bodell W.J., Aida T., Berger M.S., Rosenblum M.L. Increased repair of O6-alkylguanine DNA adducts in glioma-derived human cells resistant to the cytotoxic and cytogenetic effects of 1,3-bis(2-chloroethyl)-1-nitrosourea. Carcinogenesis 7:879-883(1986)

12.- Rutka J.T., Giblin J.R., Dougherty D.Y., Liu H.C., McCulloch J.R., Bell C.W., Stern R.S., Wilson C.B., Rosenblum M.L. Establishment and characterization of five cell lines derived from human malignant gliomas. Acta Neuropathol. 75:92-103(1987)

13.- Nister M., Westermark B. Human glioma cell lines. (In) Atlas of human tumor cell lines; Hay R.J., Park J.-G., Gazdar A.F. (eds.); pp.17-42; Academic Press; New York (1994)

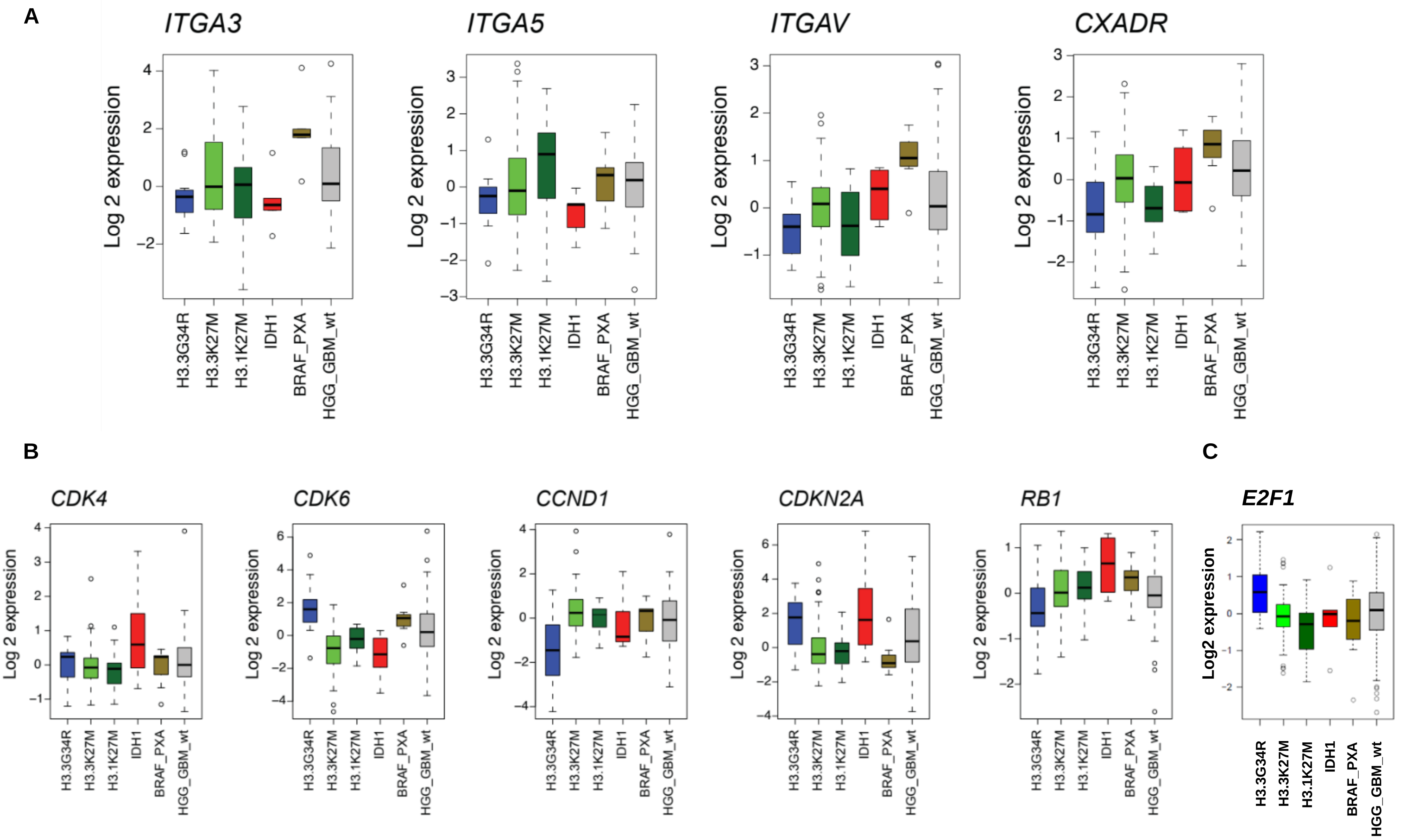

Supplementary Figure 1. *In silico* analyses of Delta-24-RGD determinants of replication and infection in pHGG and DIPGs patients. (A) Assessment of the expression of integrins and CAR in pHGG and DIPGs (B) Quantification of the expression of RB pathway genes in pHGG and DIPGs (C) Evaluation of E2F1 expression in pHGG and DIPG**.**

Supplementary Figure 2

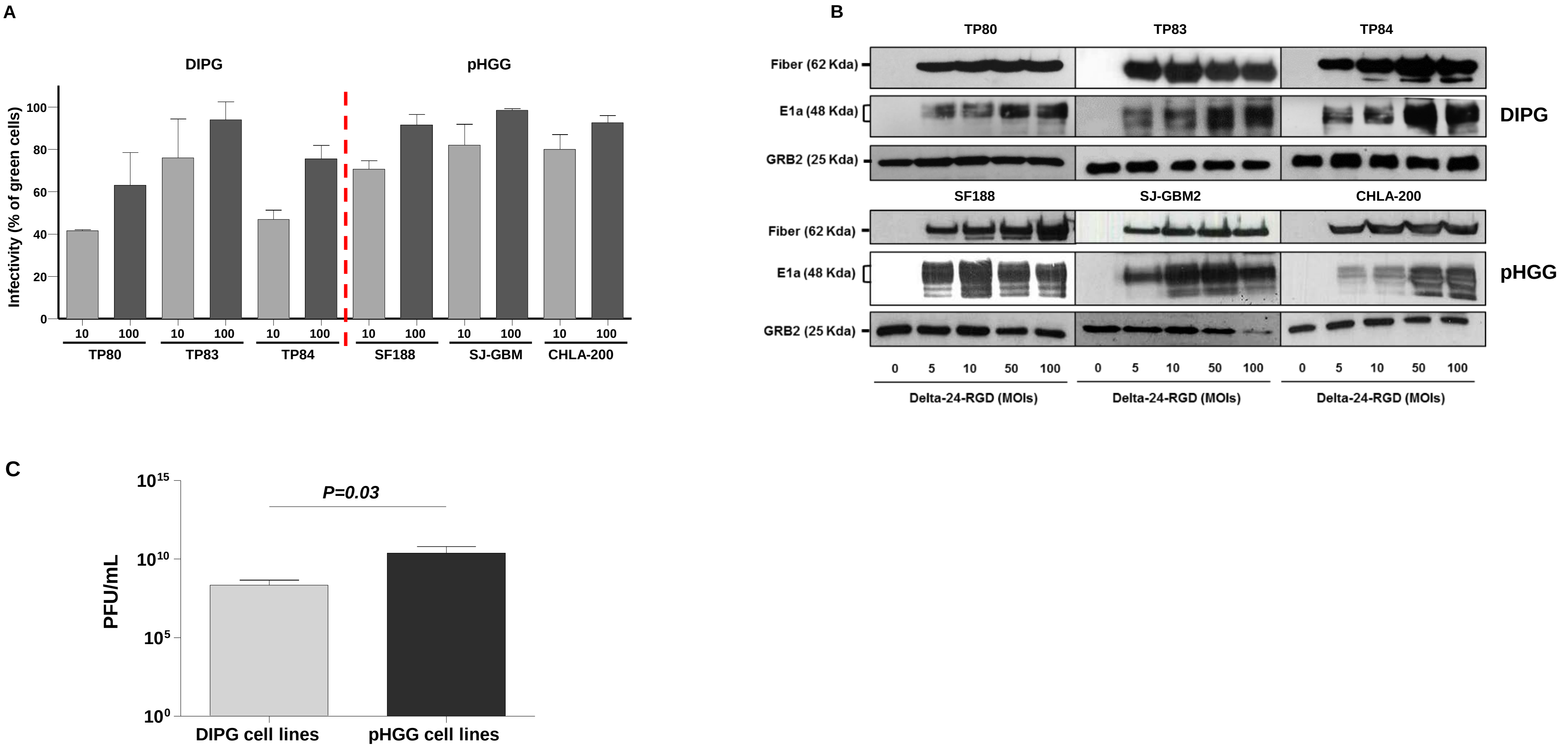

Supplementary Figure 2. DIPG and pHGG cell lines are susceptible to Delta-24-RGD infection and replication. (A) Flow cytometry analyses of infectivity in DIPG and pHGG cell lines. The indicated cells lines were infected at 10 and 100 MOIs with a replication-deficient construct expressing a modified fiber knob (AdGFP-RGD). The data are shown as the relative percentage (mean ± SD) of GFP-positive cells scored among 10,000 cells per treatment group. (B) Assessment of viral protein expression in pediatric glioma cell lines infected with Delta-24-RGD by western blotting. A representative blot of three independent experiments is shown. (C) Comparison between the media of the viral replication in DIPG versus pHGG cell lines. Viral titers were determined 3 days after infection at an MOI of 10 by an anti-hexon staining-based method in 293 cells and expressed as plaque-forming units (pfu) per milliliter. The data are shown as the mean ± SD of three independent experiments (shown in Fig. 2D).

Supplementary Figure 3

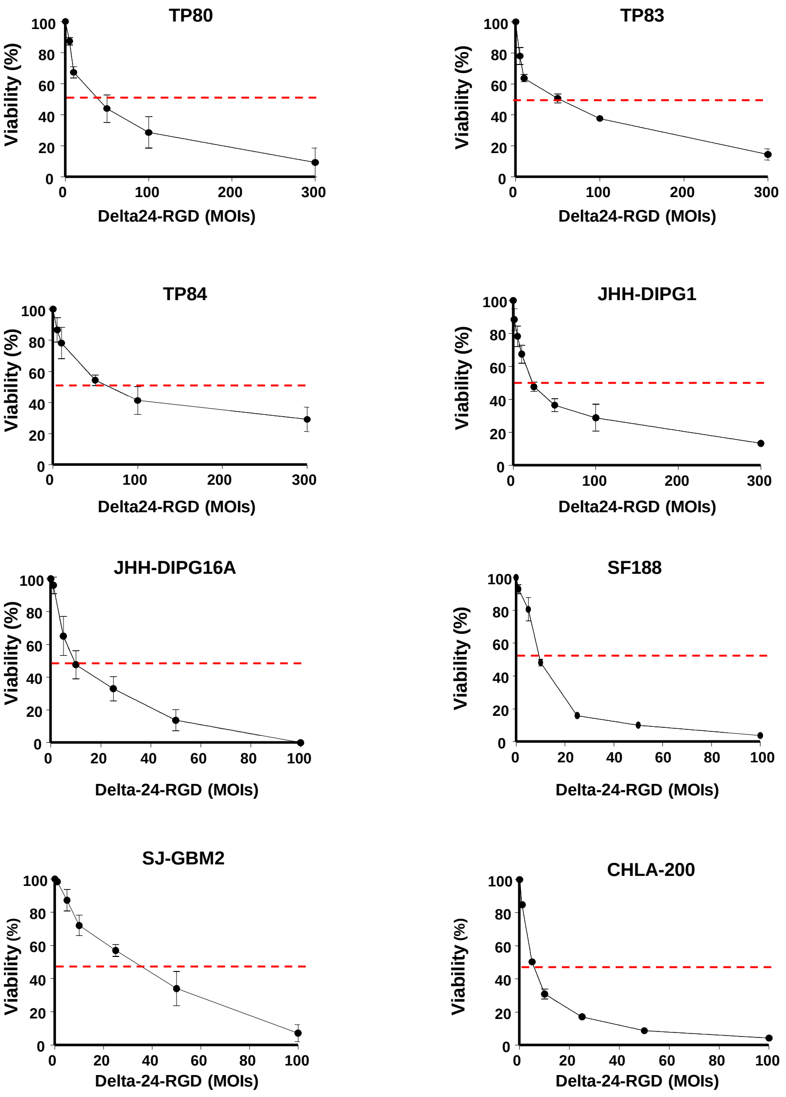

Supplementary Figure 3. Delta-24-RGD exerts a potent oncolytic effect in DIPG and pHGG cell lines. Cell proliferation analyses of Delta-24-RGD-infected DIPG and pHGG cell lines. Cell viability was assessed 5 days after infection. The data are shown as the percentage (mean ± SD of three independent experiments) of cells alive after infection with Delta-24-RGD at the indicated multiplicities of infection (MOIs) relative to the non-infected cells (control, equal to 100%).

Supplementary Figure 4

A

			Survival (Days)	*P* value
**DIPG**	**TP80**	Control	217	0.024
		Delta-24-RGD	251	
	**TP54**	Control	83.5	0.04
		Delta-24-RGD	95.5	
**pHGG**	**CHLA-03-AA**	Control	46	<0.0001
		Delta-24-RGD	99	
	**PBT-24**	Control	75	0.0013
		Delta-24-RGD	100.5	

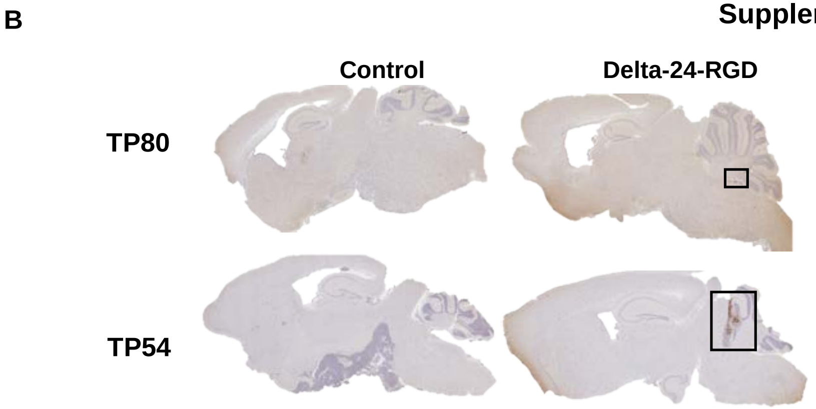

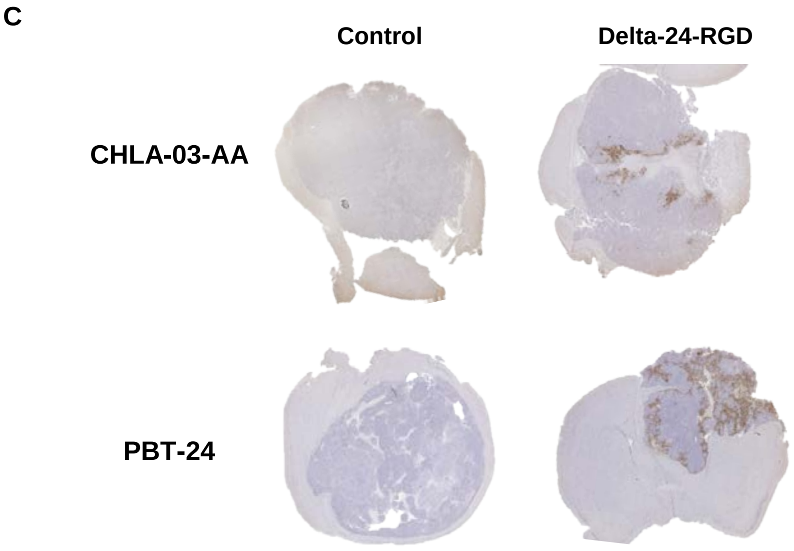

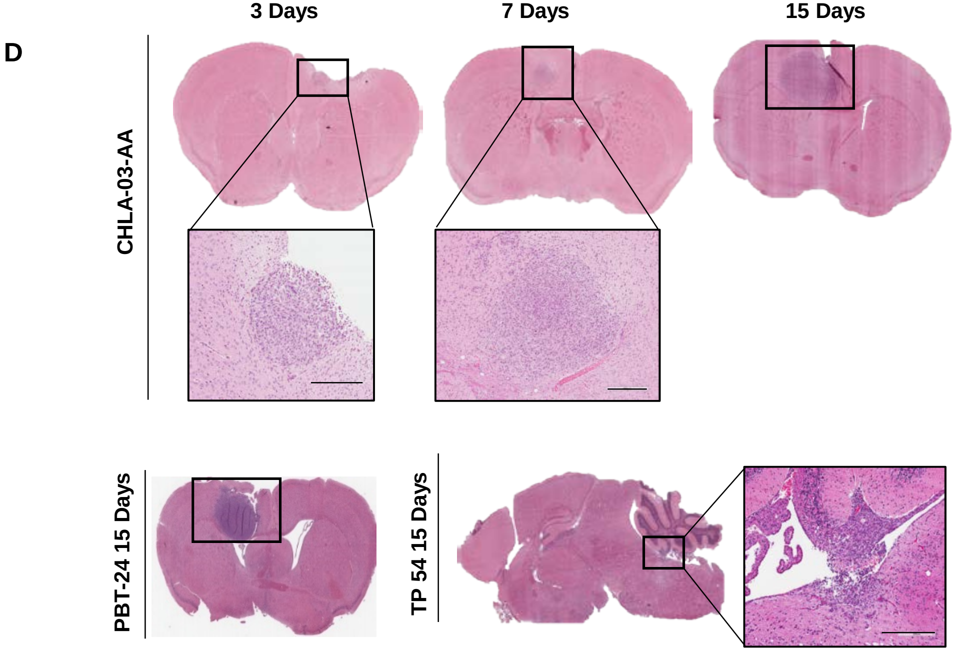

Supplementary Figure 4. Delta-24-RGD increases the overall survival in pHGG and DIPG models. pHGG. (A) Table summarizing the median survival average and log-rank test P-value of treatment groups from each experiment. (B and C) Representative low-amplified images of hexon positive staining of PBS-treated or Delta-24-RGD-treated DIPG (B) and pHGG (C) tumors. Mice were treated with either PBS (control) or Delta-24-RGD 3 days post-cell injection. For comparison studies, analyzed brains are derived from mice that died at a similar time point from control (PBS) and virus-treated groups: CHLA-03-AA= 50±10 days; PBT-24= 60±5 days; TP80= 200±5 days; TP54= 90±5 days. (D) Representative H&E images of mice brain recollected at the indicated time points (3, 7 and 15 days) illustrating tumor implantation of CHLA-03-AA, PBT-24 and TP54 cell lines.

Supplementary Figure 5

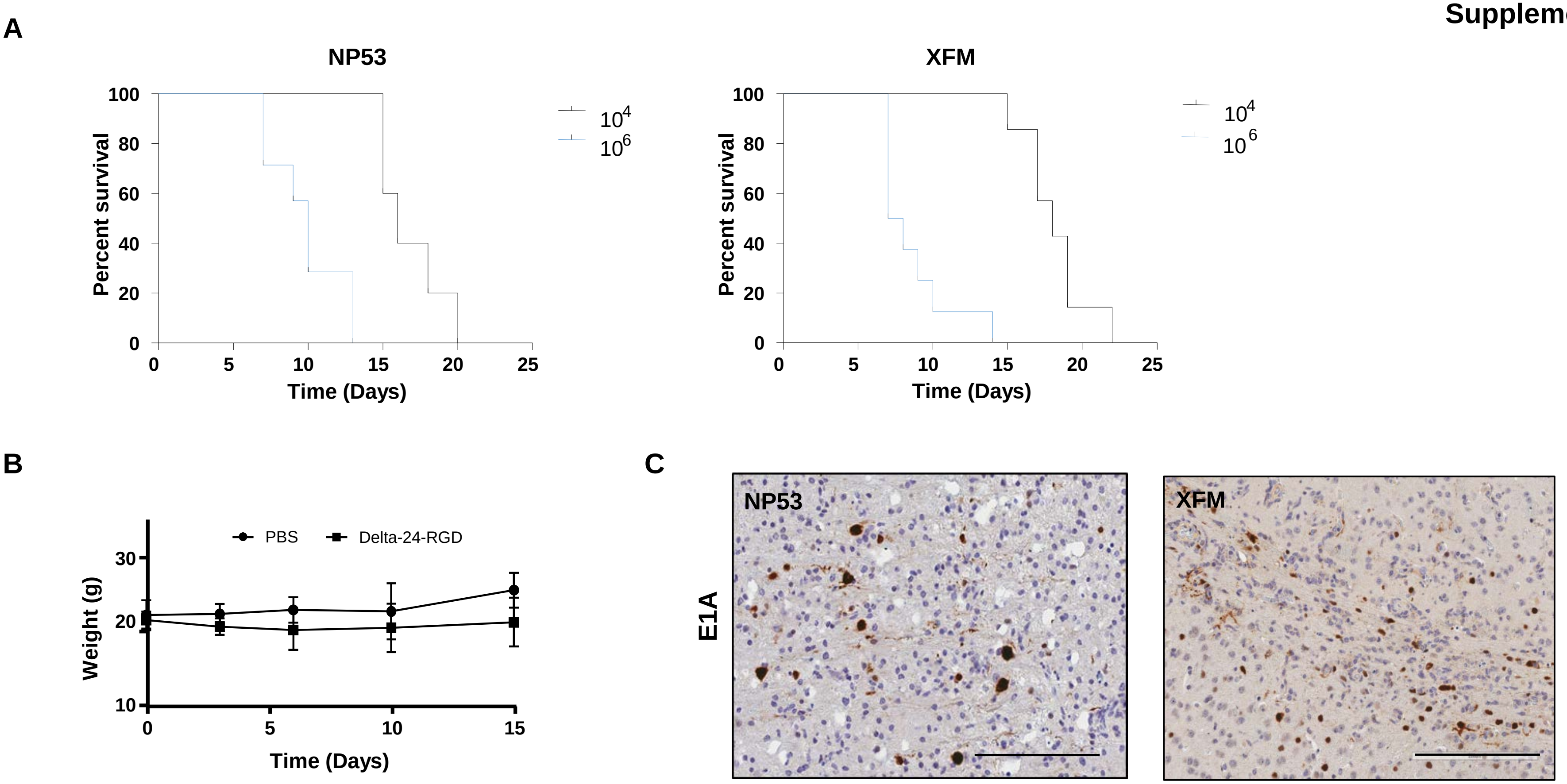

Supplementary Figure 5. Immunocompetent model characterization. (A) Kinetic analyses of NP53 and XFM. Kaplan-Meier survival curves of the indicated immunocompetent mice bearing NP53 or XFM (10^4 or 10^6) cells. (B) Brain tumors were developed by intracranial injection of the NP53 cell line, and PBS or Delta-24-RGD were administered intratumorally 3 days after cell implantation and animals were sacrificed at day15 after cell implantation. Body weight plot of non-treated and treated animals during the 15 days of the experiment. Mice were weighed every 3 or 4 days. The data are shown as the median ± SD of each measure in each group. (C) Illustrative E1A positive images of NP53 and XFM tumors treated with Delta-24-RGD. Scale bars correspond to 200nm.

Supplementary Figure 6

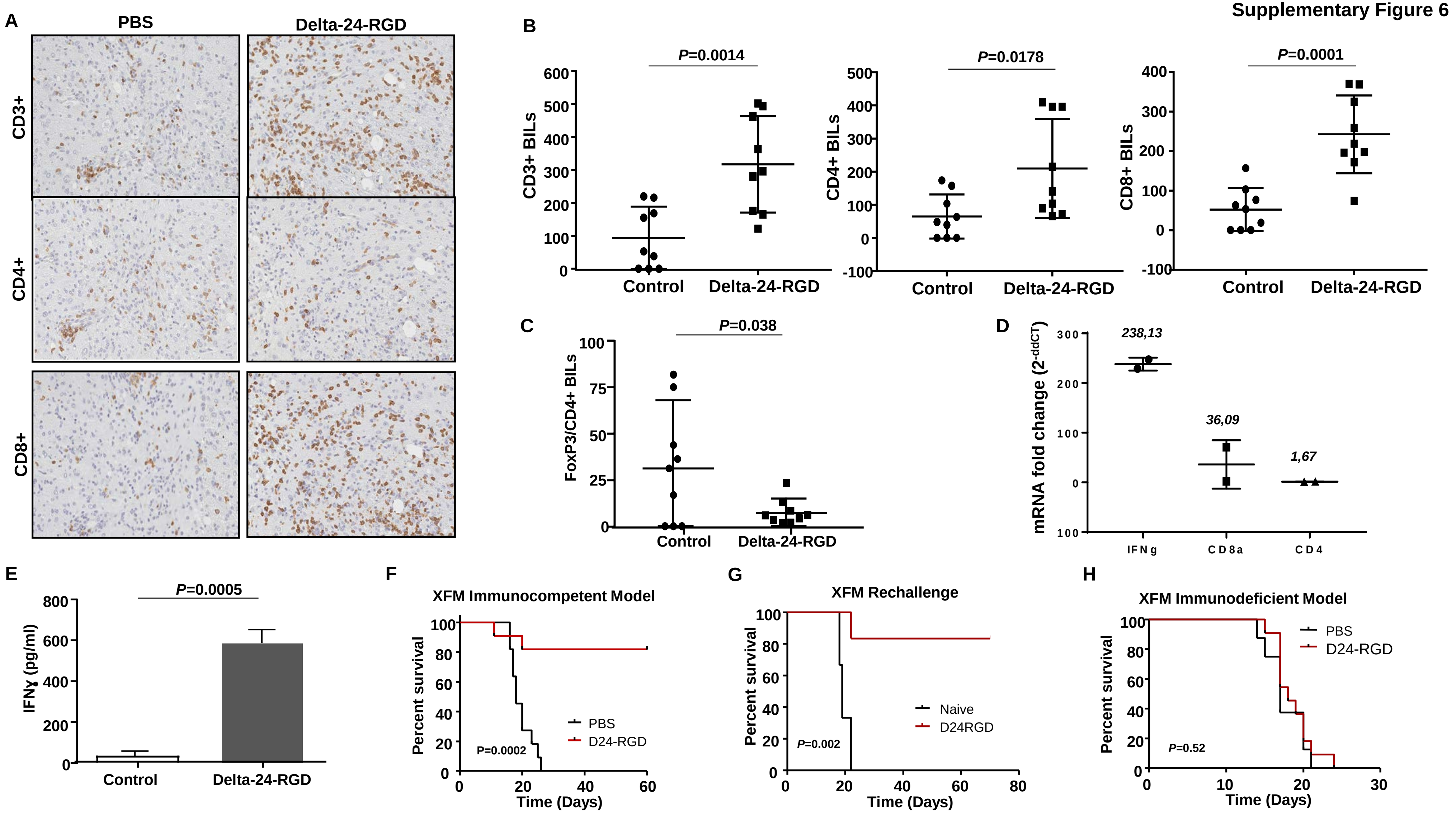

Supplementary Figure 6. Delta-24-RGD triggers a strong immune response in an XFM midline glioma immunocompetent model. Tumors were developed by intracranial injection of XFM cell line, and PBS or Delta-24-RGD was administered intratumorally on days 3, 5 and 7 post-cell injection and animals were sacrificed at days 15 to evaluate the immune response to the virus (A-E). (A) Representative images showing CD3, CD4 and CD8 infiltration in the brain after viral or PBS administration. Brains were analyzed 15 days after cell implantation. (B) Quantification of CD3, CD4 and CD8-positive cells in mouse brains (N=3) treated with PBS or Delta-24-RGD. The data are shown as positive cells counted in three different brain areas (Student's t-test). (C) Quantification of FoxP3+ cells infiltrated in the brain after the treatment administration. The data are shown as the percentage of CD4+FoxP3+ cells, counting all CD4-positive cells as 100%. (D) Quantification of mRNA expression levels in DIPG tumors (N=2). IFN gamma (mean fold change 238), CD8a (mean fold change 36.09) and CD4 (mean fold change1.67) data are shown as mRNA fold change normalized to the mRNA expression in the PBS-treated tumors. (E) Quantification of IFN gamma production in splenocytes extracted from PBS or Delta-24-RGD-treated mice that were co-cultured with XFM cells. (F) Kaplan-Meier survival curves of Delta-24-RGD (10^7 pfu)- and control (PBS)- treated immunocompetent mice (Balb/C) (N=11) bearing XFM tumors. (G) Rechallenge experiment of the long-term survivors from (F). Six long- term survivors from the Delta-24-RGD treated group were subjected to a rechallenge with XFM cells and compared with control untreated mice (N=6; both groups). (H) Kaplan-Meier survival curves of Delta-24-RGD (10^8 pfu)- and control (PBS)- treated immunocompromised mice (athymic nude) (n=11) bearing intracranial XFM tumors.
